# Automated T2* maps of the heart and liver in comparison to manual analysis for iron overload assessment in the All Iron Detected (AID) multicenter study

**DOI:** 10.1186/1532-429X-17-S1-W18

**Published:** 2015-02-03

**Authors:** Juliano L Fernandes, Maria Helena A Siqueira, Karina T Nobrega de Oliveira, Luiz Francisco Avila, Ilan Gottlieb, Marly U Lopes, Andre M Fernandes, Ralph Strecker, Andreas Greiser

**Affiliations:** 1 University of Campinas, Campinas, Brazil; 2 Cardiovascular Imaging, Jose Michel Kalaf Research Institute, Campinas, Brazil; 3 Santa Joana Diagnostico, Recife, Brazil; 4 CDPI, Rio de Janeiro, Brazil; 5 Cardiovascular Imaging, DASA, Sao Paulo, Brazil; 6 Hospital Ana Nery, Salvador, Brazil; 7 Siemens Ltda, Sao Paulo, Brazil; 8 Healthcare, Siemens AG, Erlangen, Germany; 9 Radiologia Clinica de Campinas, Campinas, Brazil; 10 Hospital Mater Dei, Belo Horizonte, Brazil; 11 Hospital Sirio Libanes, Sao Paulo, Brazil

## Background

Estimation of heart and liver iron concentrations using T2* has become an essential diagnostic step in the management of iron overload. One of the limitations of more widespread use of these techniques has been calculating the T2* values from the original images due to need of special training and dedicated softwares. We sought to assess whether the use of an automated T2* in-line calculation protocol applied in a multicenter study in centers with different initial experience in this technique would be feasible and accurate.

## Methods

Seventy-six patients of the multicenter study All Iron Detected (AID) involving seven centers using different 1.5T scanners from a single vendor (Siemens AG) were included. Automated T2* maps were calculated in-line based on a prototype evaluation using original multi-echo images of the heart and liver with values obtained by drawing a region of interest in each organ directly in the originated map. Manual calculation of these values were also obtained by fitting the decay curves of the original images using the truncation method both locally as well as in a central core lab. Sixteen patients had repeated scans using a routine protocol with manual evaluation for comparison. Values of the automated pixel-wise fit T2* of the heart and liver were compared to the local manual ROI-fitting technique as well as the central core lab results values using Bland-Altman plots and intraclass correlation coefficients (ICC). Inter-scanner coefficients of variation among the repeated scans using the automated versus manual calculations was also assessed.

## Results

The mean age of the patients was 32.1±23.1 years (55% male) with median automated T2* values of 31.1ms (range 4.2 to 61ms) for the heart and 5.5ms (range 0.7 to 32.4ms) for the liver. In the heart, the automatically calculated T2* values were significantly correlated to both local and core lab manual calculations, with a mean difference of -2.0ms (95%CI -2.9 to -1.1 ms) for the core lab comparison and ICC of 0.95 (95%CI 0.89 to 0.97). In the liver, the mean difference was 0.18ms (95%CI -0.03 to 0.39ms) with ICC of 0.99 (95%CI 0.991 to 0.996) comparing automated versus core lab manual measurements despite a lesser degree of agreement in patients with severe liver iron concentration (T2* < 1.8ms) with 0.5ms (95%CI 0.23 to 0.80ms) and ICC of 0.22 (-0.35 to 0.64). In the inter-scanner comparison, the coefficient of variation among the subgroup tested was 12.8% for the heart and 14.1% for the liver.

## Conclusions

The use of automated T2* maps for iron overload assessment of the heart and liver is feasible across different centers and results in accurate results compared to the traditional manual evaluation by a central core lab.

## Funding

N/A.

